# Exploring Profiles of Risk and Protective Factors Among Youth Mentees: For Whom Does Mentoring Work?

**DOI:** 10.1007/s11121-026-01878-3

**Published:** 2026-01-16

**Authors:** Margaret Meldrum, Michael D. Lyons

**Affiliations:** School of Education and Human Development, University of Virginia, 405 Emmet Street S, Charlottesville, VA 22903 USA

**Keywords:** Youth mentoring, Risk, Resilience, Positive youth development

## Abstract

Youth mentoring programs are an increasingly popular intervention and prevention strategy to promote positive youth development and to address a range of youth needs. Past research shows positive, albeit moderate, effects of mentoring across multiple domains, but there is evidence that suggests heterogeneity in treatment outcomes. Several studies have examined the role of risk factors in mentoring outcomes, but less is known about the role of protective factors. This study examines the extent to which ecological factors outside of the mentoring relationship, specifically, youth risk factors and existing social support, play a role in the effectiveness of mentoring in promoting adaptive coping outcomes, as measured by academic achievement, self-efficacy, and expectations. Using a person-centered approach, we examined (1) whether there were distinct profiles of youth participating in mentoring using mentee risk factors and existing social support as indicators; (2) associations between profiles and youth race/ethnicity and gender; and (3) whether profiles differed in post-program adaptive coping outcomes. Two classes of youth were identified. One class reported higher risk factor presence and higher social support and was more likely to be youth of color. The second class reported lower risk factor presence and lower social support. Classes did not differ in their adaptive coping outcomes. The implications of these findings for mentoring programs and further research are discussed.

Mentoring programs are popular interventions that pair youth with a caring, non-parental adult to promote healthy development. Mentoring has been implemented to support a range of youth needs and populations based on empirical work that has demonstrated its effectiveness in promoting improvements across social, emotional, academic, and behavioral outcomes (DuBois et al., 2002, 20l1; Herrera et al., 2013; Raposa et al., 2019). Despite research showing small, positive average effects, there is some evidence that suggests heterogeneity in treatment outcomes (Lyons & McQuillin, 2019a; Lyons et al., 2019b). Specifically, the effectiveness of mentoring may depend on the quality of and the activities that occur within the mentoring relationship (Cavell et al., 2021) as well as ecological factors outside of the relationship, including mentee risk and protective factors (Sánchez et al., 2018). Consideration of the contexts and characteristics that may lead to developmental challenges (i.e., risk factors) and those that may offset the effects of risk factors (i.e., protective factors) to promote positive adaptations (Spencer, 2006a, b, 2008, 2024) expands mentoring’s traditional emphasis on risk reduction. The current study sought (a) to identify and characterize youth profiles of risk factors and existing social supports upon entry into mentoring and (b) to test whether the addition of a mentor as a social support has a differential impact by profile on adaptive coping outcomes, as measured through academic self-efficacy, academic performance, and educational aspirations.

## Theoretical and Empirical Background

Studies of youth mentoring often look at how individual risk factors and, less frequently, protective factors may moderate program effectiveness. For example, DuBois et al. (2011) found that program effects were stronger for those serving youth with heightened individual risk (e.g., academic or behavioral challenges) but low environmental risk or for youth with heightened environmental risk but low individual risk. However, theories of youth development suggest there may be greater complexity in assessing youth risk and protective factors. Specifically, such factors may have an interactive effect; while risk factors may predispose youth to adverse outcomes, protective factors may offset these to promote positive outcomes (Sánchez et al., 2018; Spencer et al, 1995, Spencer, 2024). For example, social support (Cooper et al., 2013; Griffith et al., 2019) and racial/ethnic socialization (Anderson & Stevenson, 2019) may offset some of the deleterious effects of racial discrimination for youth. To add additional complexity to these models, researchers have also suggested that youth’s social identities (e.g., race, gender) may further complicate how risk and protective factors relate to outcomes in mentoring. Specifically, the extent to which risk and protective factors manifest as challenges or support is dependent on an individual’s perceptions of such factors and is affected by systemic and structural forces that confer privilege or marginality (e.g., systemic racism, patriarchal norms) (Spencer, 2024).

### The Phenomenological Variant of Ecological Systems Theory

The *Phenomenological Variant of Ecological Systems Theory* (PVEST) is a helpful framework to conceptualize how risk and support may lead to differential benefits of youth mentoring. PVEST describes the bidirectional relationships between *net vulnerability* (i.e., risk and protective factors), *net stress* (i.e., challenges confronted and social support), and *reactive coping processes* (i.e., adaptive and maladaptive), and how each is related to youth’s emergent identities and developmental outcomes (Spencer et al, 1995). Additionally, this framework includes systemic and structural forces at multiple ecological levels that shape youth development (Spencer, 2024; Spencer & Tinsley, 2008). Importantly, PVEST highlights that although youth may have the same risk factor, the extent to which it is experienced as a challenge differs across individuals due to the presence of protective factors and whether these factors are perceived by youth as an accessible support.

**Fig. 1:**
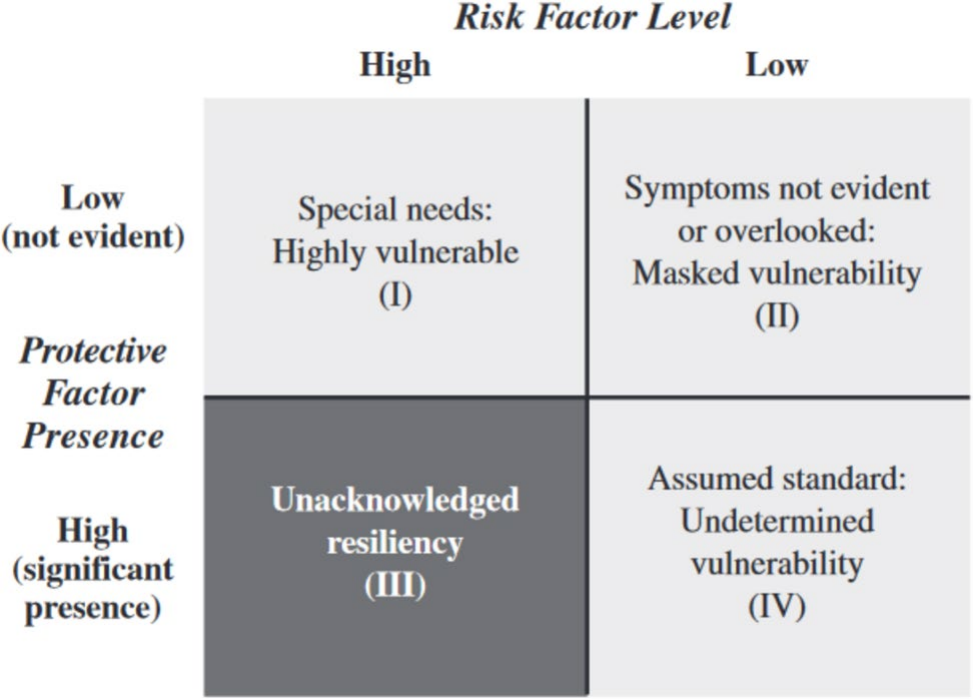
Margaret Beale Spencer’s Dual-Axis Coping Formulation of PVEST (Source: “Phenomenology and Ecological Systems Theory: Development of Diverse Groups” pp. 829–893, by M. B. Spencer, in *Handbook of Child Psychology: Vol. 1. Theoretical Models of Human Development*, *Sixth Edition*, W. Damon (Series Ed.) & R. Lerner (Vol. Ed), 2006, Hoboken, NJ: Wiley)

The *Dual-Axis Coping Formulation of PVEST* has been posited as a means of exploring youth profiles of net vulnerability as they relate to coping, identities, and developmental outcomes (Spencer, 2006a, b). This formulation conceptualizes risk and protective factors as falling along a continuum across two perpendicular axes (Fig. 1). Youth with high risk and low protective factor presence are considered “highly vulnerable” and are assumed to have high needs. Youth with low risk and low protective factor presence are considered to have “masked vulnerability” because their needs are not immediately apparent. Youth with high risk and high protective factor presence are considered to have “unacknowledged resilience,” often demonstrating positive adaptations in the face of challenges, yet, their resilience, unfortunately, can be overlooked due to their high-risk status. Those with low risk and high protective factor presence are considered to have “undetermined vulnerability” and are often conceptualized as normative in developmental literature. The extent to which mentees reap differential benefits from youth programming by profile has not been tested.

No studies known by the authors have applied PVEST as a guiding framework to examine youth mentoring effects. However, in a recent study of youth-adult relationships, PVEST was used to explain how a positive relationship with a non-parental adult served a protective function for youth who were exposed to community violence (Wilson et al., 2025). The orientation of many mentoring programs—to leverage the strengths and assets of youth and their communities to promote healthy development—coupled with the tendency for recruitment to target young people considered at risk due to the presence of multiple risk factors, creates a fruitful context for examining net vulnerability and whether outcomes vary by net vulnerability, using PVEST as a guiding orientation (Spencer, 2006a, b, 2008). Moreover, protective factors and adaptive coping responses to inequitable conditions are underexamined among Black and Brown youth due to an overemphasis on pathology and risk among these youth because of systemic racism, holding implications for how policies and practices are developed and implemented (Spencer, 2024). Without a clear understanding of how both risk and support are implicated in the effectiveness of youth programming (i.e., mentoring) for *all* youth, it is difficult to provide the right level and type of intervention for youth.

### Youth Risk Factors

Youth socioeconomic status (SES) and family structure and dynamics are often conceptualized as risk factors. Family instability, such as living in a single-parent home or parent incarceration, and disruptions to youth’s caregiving system, such as foster care placement, have been linked with poorer social, emotional, psychological, and behavioral outcomes (Harden, 2004; Haskins et al., 2018; Manning & Lamb, 2003). For example, youth who experience disruptions to their primary caregiving systems exhibit more internalizing and externalizing symptoms (Poon et al., 2022). Family dynamics, such as conflict and emotional support, which may be even more important than family structure (e.g., parents’ marital status) in some instances, also play a role in children’s development (Umberson & Thomeer, 2020); for example, family conflict contributes to increased internalizing and externalizing symptoms among children (Jones et al., 2021). Moreover, those from low-income homes may have less access to resources, healthcare, and other services and support due to structural barriers, which can contribute to mental and physical health challenges (Condliffe & Link, 2008; Costello et al., 2003; Faruque et al., 2025). The interplay between race/ethnicity and SES in the United States increases the likelihood that youth in racialized and under-resourced communities will experience a stressful or traumatic event (Lieberman et al., 2011). Low-income neighborhoods are also overpoliced, and youth behaviors are more likely to be interpreted as deviant or criminal, as opposed to adolescent or juvenile, increasing the likelihood of contact with the legal system (Goff et al., 2016; Sampson, 1986).

Racial–ethnic identities and gender are widely studied in mentoring research (Chesney-Lind et al., 2008; De Los Reyes et al., 2022; Dove, 2022; Gowdy et al., 2024; Jones et al., 2023; Marks et al., 2020; Simpson et al., 2023; Spencer et al., 1996). These identities place youth in a marginalized position due to structural inequities that make it more likely that they will encounter challenges associated with racism and/or sexism (Marmot & Wilkinson, 2005). Within the PVEST framework, social identities (i.e., race, ethnicity, gender) are part of an individual’s net vulnerability that may or may not lead to experienced challenges (i.e., net stress). With respect to racial or ethnic identity, oppression and segregation create a context in which youth are more likely to experience environmental challenges, such as community violence (Archambault et al., 2017; Center for the Developing Child, 2019; Pearce et al., 2010). Structural racism and inequitable policies further contribute to public health disparities, economic marginalization, and inequitable access to education, healthcare, housing, and employment (Marks et al., 2020). Although there is not consistent evidence that mentoring outcomes differ by youth race or ethnicity (e.g., Herrera et al., 2013; Raposa et al., 2019), these circumstances warrant consideration of race/ethnicity when examining youth risk and protective factors. With respect to gender, youth face heightened pressures to conform to dominant norms of masculinity and femininity at distinct points in development, which are linked with maladaptive coping and negative outcomes (Brown & Gilligan, 1992; Chu & Gilligan, 2014; Way, 2011). For boys, gender norms can encourage emotional stoicism, hyper-independence, and physical aggression, which can lead to a loss of social support (Way, 2011, 2024). For girls, gender norms include pressures to not voice their needs in relationships (Brown & Gilligan, 1992; Gilligan & Snider, 2018), which may result in a loss of connection (Brown & Gilligan, 1992; Hipwell & Loeber, 2006; Rhodes et al., 2008; Taylor et al., 1995; Williamson et al., 2020). Given the origins of mentoring as a means of preventing delinquency among boys (DuBois, 2021), programs may not attend to girls’ needs (DuBois et al., 2011; Raposa et al., 2019). To this point, in some cases, adolescent boys have responded better to mentoring than girls (DuBois et al., 2011; Raposa et al., 2019). Thus, these identities are important to attend to when evaluating the effectiveness of mentoring programs. Meta-analytic work conducted over the last few decades has evidenced mixed findings regarding the role of risk in mentoring outcomes. Several studies show that youth with heightened environmental risk factors (e.g., living in poverty, neighborhood violence exposure) (DuBois et al., 2002) and individual challenges (e.g., delinquent behavior, school discipline) (DuBois et al., 2011) reap greater benefits from mentoring. Youth who have been exposed to recent stressful or traumatic events have also reported more positive mentoring outcomes (Herrera et al., 2013). Other studies have shown varied effects of youth risk depending on the outcomes of interest (Herrera et al., 2013). More recently, a meta-analysis of mentoring programs found consistent effects across outcomes for youth with varied levels of risk (Raposa et al., 2019). Notably, for youth with very high risk across domains, more comprehensive intervention may be needed (Herrera et al., 2013).

### Youth Protective Factors

Protective factors are the contexts or characteristics of a youth’s environment that can lower the likelihood of negative outcomes and raise the likelihood of positive outcomes (Spencer et al., 2004). Social support, including the presence of peers, caregivers, and other significant adults, is routinely identified as an important protective factor that can contribute to positive adaptations, or resilience, in the face of risk when they are accessible and experienced as supportive. For example, positive youth-caregiver relationships and friendships can lessen the stress and negative impact of racism on behavioral and mental health outcomes (Brenner et al., 2016; Brody et al., 2006) and are predictive of less delinquent behavior (Jacobsen & Zaatut, 2020). Likewise, negative youth-caregiver relationships can have a negative impact on healthy development (Ranson & Urichuk, 2008; Umberson & Thomeer, 2020) and have been linked with delinquency (Hoeve et al., 2009). Peer relationships are an increasingly important source of support as youth move through adolescence. Youth with multiple risk factors who have high-quality social connections have shown more positive adaptations across academic, behavioral, and psychosocial domains (Li et al., 2011; Vanderbilt-Adriance et al., 2015; Wentzel et al., 2021), and these positive relationships may buffer the sequelae of adverse or traumatic experiences (Coatsworth, 1998; Chapin, 2014; Easterbrooks et al., 2024; Kim-Cohen et al., 2004; Lerner et al., 2013; Lieberman et al., 2011; Luthar et al., 2000; Masten & Pellegrini, 1990; Scheeringa & Zeanah, 2001). Youth mentoring programs are thought to provide youth with an additional form of social support to buffer against risk factors (Hrdy, 2009; Li & Julian, 2012). However, mentees who experience social challenges may struggle to form close bonds with their mentors and may not necessarily experience mentors as a source of support (Poon et al., 2022; Rhodes, 2005; Williamson et al., 2020).

Natural, or informal, mentoring relationships are also a protective factor and potential source of support for youth (Bowers et al., 2015; Deutsch et al., 2020. Unlike formal mentors, these relationships develop organically between youth and adults in their pre-existing social networks (e.g., relatives, coaches, teachers, after-school staff, and neighbors). The presence of a natural mentor can contribute to improved academic (Culyba et al., 2016; Gordon et al., 2009) and vocational functioning (Van Dam et al., 2018), socioemotional development, and psychosocial outcomes (Van Dam et al., 2018), as well as serve a protective function in the face of racial discrimination (Cooper et al., 2013). Like formal mentors, the quality and connectedness of the natural mentoring relationship are important for promoting positive outcomes (Hurd & Seller, 2013; Van Dam et al., 2018). Recent meta-analytic work indicates that youth can reap the benefits of these relationships independent of several risk factors (Van Dam et al., 2018).

### Adaptive Coping

Academic self-efficacy, academic achievement, and educational aspirations are generally agreed upon as proxies for adaptive coping (Spencer et al., 2004) and are common outcomes targeted by mentoring programs due to their association with other positive cascading effects (Spencer et al., 2004; Tanner-Smith et al., 2018; Tolan et al., 2014). Past research has shown that mentors can promote positive self-views (e.g., self-efficacy) and academic performance for mentees (Grossman et al., 2012; Herrera et al., 2011), while risk factors can negatively impact youth’s academic performance and self-views (Spencer et al, 2004). Maintaining a positive self-view, including high self-efficacy and aspirations in the face of heightened challenges, is indicative of adaptive coping and contributes to more positive academic performance (Jung & Zhang, 2016; Khattab, 2015; Otani, 2020). High academic achievement is associated with reduced risk behaviors and better long-term outcomes, while low achievement, disengagement, and truancy are precursors to greater experienced challenges and delinquency (Bradley & Greene, 2013; Fiscella & Kitzman, 2009; Hirschfield & Gasper, 2011; Rocque et al., 2017; Topitzes et al., 2009).

### The Current Study

The consistently modest effects of mentoring that are reported in the literature warrant further research about for whom mentoring is the best form of intervention and under which circumstances, considering risk factors and social support. Few studies have examined how both risk and protective factors concurrently play a role in mentoring outcomes. Accordingly, we addressed three research questions. First, what are the profiles of risk factors and existing social supports among adolescents participating in one-on-one youth mentoring upon program entry? Drawing on the *Dual-Axis Coping Formulation of PVEST*, we expected profiles to vary along a continuum for risk factor presence and social support. We then examined whether profiles were associated with race/ethnicity or gender. Finally, using the predicted profiles, we examined whether the addition of a mentor—an added social support—led to differential benefits for youth in academic self-efficacy, academic achievement, and educational aspirations—conceptualized as proxies for adaptive coping or resilience. Past research has demonstrated that youth with very heightened risk factors across domains benefit less from mentoring in terms of academic performance, as these individuals may need a greater degree of support than a mentor can provide (DuBois et al., 2011; Raposa et al., 2016; Schwartz et al., 2011). Therefore, we expect youth with a moderate degree of risk, including those with lower risk factor presence and lower social support and those with high risk factor presence and high social support, to benefit most from mentoring.

## Methods

This study was approved by the Institutional Review Board at the academic institution with which the research team is affiliated. Data were collected between 2014 and 2018 as part of routine operating procedures to support continuous quality improvement efforts of Big Brothers Big Sisters of America mentoring program (BBBSA). BBBSA administered surveys to mentees across sites in the United States upon each mentee’s entrance into mentoring (baseline) and for at least one follow-up timepoint, approximately 1 year or one school semester after the baseline data collection timepoint (follow-up). Mentees provided background demographic information at baseline. Prior to survey completion, the mentees’ guardians provided informed consent.

Participants include youth mentees who participated in a BBBSA mentoring program at sites across the United States and fully completed a baseline survey and a follow-up survey within 9–16 months (*N* = 494). The average age of mentees at baseline was 15.8 years (*SD* = 2.4; range, 11–24 years), with 60.1% identifying as female and 39.9% identifying as male. In terms of race-ethnicity, 48.6% of mentees identified as White, 18.0% identified as Black, 13.6% identified as Hispanic, 2.2% identified as American Indian/Native Alaskan, 0.8% identified as Asian, 12.8% identified as two or more races, and 4.0% identified as other. Due to small sample sizes, youth were categorized as White or Youth of Color for analysis. Most mentees participated in community-based mentoring programs (87.7%) (Table 1). Our sample was similar to the general population of BBBSA mentees in terms of age and gender but differed in terms of race-ethnicity; more Youth of Color are served in the general BBBSA population relative to our sample (Mahoney & Fain, 2023).

**Table 1:** Participant and program characteristics

Category	*n* (average)	% (*SD*)	Category	*n*	%
Age	(15.77)	(2.39)	FRL		
Gender			Yes	401	81.2
Male	197	39.9	No	93	18.8
Female	297	60.1	Parent incarcerated		
Race/ethnicity			Yes	104	21.1
White	240	48.6	No	390	79.0
Black	89	18.0	Living situation		
Hispanic	67	13.6	One parent	327	66.2
American Indian or Alaskan Native	11	2.2	Two parents	121	24.5
Asian	4	0.8	Other relative^1^	46	9.3
Two or more races	63	12.8	Special adult		
Other	20	4.0	Yes	383	77.5
Program type			No	111	22.5
Community	433	87.7			
School	61	12.3			

^1^Unknown, foster care, group home, institution, other relative, or other

### Measures

#### Sociodemographic Characteristics

BBBSA collected and reported data regarding mentee sociodemographic characteristics, including mentees’ race/ethnicity, gender, age, living situation (foster home, grandparents, group home, institution, one parent (specified female or male), other relative, other/unknown, sibling guardian, two parents (specified two mothers, two fathers, marital status)), whether a mentee had an incarcerated parent, and whether a mentee received free-or-reduced-price lunch (FRL). The living situation was re-coded into two parents (any combination of male or female and marital status), one parent (either male or female), or other due to small sample sizes. See Table 1 for a summary of sociodemographic characteristics.

#### Social Support

Mentees’ perceptions of their existing social support (peers, primary caregiver, and natural mentor) were reported using three subscales.

##### Peer Support

Peer support was measured using a 6-item measure adapted from the “social competence” subscale of the Self-Perception Profile for Children (SPPC; Harter, 1985; Herrera et al., 2023). Items have demonstrated acceptable reliability among youth mentees in this adapted format (*α* = 0.76; Herrera et al., 2023). Mentees reported their perceptions of peer social acceptance using a 4-point Likert scale with options *not at all true* (1), *not very true* (2), *sort of true* (3), or *very true* (4). Three items were positively worded (e.g., “I am always doing things with lots of kids.”) and three items were negatively worded (e.g., “I would like to have a lot more friends.”). The negatively worded items were reverse scored. Past research indicates that this scale represents two latent factors, one for the three positive indicators, with higher scores indicating higher social connectedness with peers, and one for negative indicators, with higher scores indicating social dissatisfaction (Fallavollita & Lyons, 2023). Reliability was acceptable at baseline (*α* = 0.692) and follow-up (*α* = 0.747).

##### Parent Support

Youth-caregiver relationship quality was measured using a 3-item measure adapted from the “trust” subscale of the Inventory of Parent and Peer Attachment (IPPA; Armsden & Greenberg, 1987). Mentees rated their perceptions of their relationship with their primary caregiver with whom they felt closest (e.g., “How often do I feel that my parents respect my feelings?”) using a 4-point Likert scale with options *hardly ever* (1), *not very often* (2), *sometimes* (3), and *pretty often* (4). Reliability was acceptable at baseline (*α* = 0.728) and follow-up (*α* = 0.772).

##### Natural Mentor

The presence of an important/special non-parent adult, i.e., natural mentor, was assessed using a single item: “Right now in your life, is there a special adult (not your parent or guardian) who you often spend time with? A special adult is someone who does a lot of good things for you. For example, someone (a) who you look up to and encourages you to do your best, (b) who really cares about what happens to you, (c) who influences what you do and the choices you make, and (d) who you can talk to about personal problems?” Mentees responded either *No*, *I do n*o*t have a special adult in my life right now* (0) or *Yes*, *I do have a special adult in my life* (1).

#### Adaptive Coping

Mentees indicated their perceptions of their academic self-efficacy, academic achievement, and educational aspirations using three subscales.

##### Academic Self-Efficacy

Academic self-efficacy was assessed using a 6-item subscale from the Self-Perception Profile for Children (SPPC; Harter, 1985) and a Likert-scale adaptation used by Herrera et al. (2023) (*α* = 0.68–0.64). Mentees responded to three positively worded items (e.g., “I feel that I am just as smart as other kids.”) and three negatively worded items (e.g., “I often forget what I learn.”) using a 4-point Likert scale with options *not at all true* (1), *not very true* (2), *sort of true* (3), or *very true* (4). The negatively worded items were reverse scored, and item-level responses were used to generate a mean score.

##### Academic Achievement

Academic achievement was assessed using a 4-item measure that asked mentees to report their grades in math, reading/language arts, social studies, and science. Mentees responded using a 5-point Likert-type scale with options *not good at all* (*F*) (1), *not so good* (*D*) (2), *good* (*C*) (3), *very good* (*B*) (4), and *excellent* (*A*) (5). Item-level responses were used to generate a mean score.

##### Educational Expectations

Educational expectations were measured using a 3-item measure that assessed mentees’ plans for secondary and post-secondary education. Mentees responded to items (e.g., “How sure are you that you will finish high school?”) using a 4-point Likert scale with options *Not at all sure* (1), *not really sure* (2), *mostly sure* (3), or *very sure* (4). Item-level responses were used to generate a mean score.

#### Program Variables

Program characteristics were reported for all participating youth. Program type refers to the setting of the mentoring program. This variable was created by the study team based on an iterative manual coding process. Programs that included “elementary school,” “middle school,” or “high school” in their names were coded as school-based mentoring programs, and all other programs were coded as community-based mentoring programs.

### Analytic Strategy

We used a person-centered approach to account for the dimensional nature of risk and protective factors. Specifically, we conducted a latent class analysis (LCA) to identify latent classes of mentees based on their risk factors and existing social support reported at baseline using the Mplus version 8.11 (Muthen & Muthen, 2024). Latent classes were specified using risk factors and existing social support as indicators, with youth age, youth gender, youth race/ethnicity, program type, and baseline adaptive coping measures as auxiliary variables. Model fit was evaluated using Bayes’ Information Criteria (BIC; Schwarz, 1978) and the Lo–Mendell–Rubin Adjusted likelihood ratio test (LMR-A LRT; Lo et al., 2001). When selecting the best-fitting model solution, interpretability, class size, entropy values, and substantive theory were considered (Berlin et al., 2014; Nylund et al., 2007; Schmiege et al., 2012).

Subsequently, we examined whether profiles differed with respect to youth race/ethnicity, youth gender, youth age, and program type. We predicted classes from auxiliary variables using the 3-step approach in which auxiliary variables were regressed against a selected reference class (Vermunt, 2010). Finally, we examined whether profiles differed with respect to adaptive coping at follow-up (i.e., academic self-efficacy, academic achievement, educational aspirations) using the manual BCH method (Bakk & Vermunt, 2015) while controlling for baseline adaptive coping.

## Results

### Profile Results

Results of the models tested are presented in Table 2. The 2-class solution was the best fitting model based on evaluation of fit statistics and substantive theory. The BIC value was lower in the 2-class solution, as compared to the 1- and 3-class solutions, and the LMR-A LRT *p-*value was significant for the 2-class solution, but not for the 3-class solution. Descriptive statistics for risk and support variables across classes are presented in Table 3. The first class, labeled *unacknowledged resilience* (71.1%; *n* = 343), was characterized by relatively higher risk factor presence, including lower likelihood of living in a two-parent home, greater likelihood of receiving FRL, and greater likelihood of having an incarcerated parent, and high perceived social supports, including higher perceived support from parents, greater connectedness with peers, less social dissatisfaction with peers, and higher likelihood of having a natural mentor in their life. The second class, labeled *Masked Vulnerability* (28.9%; *n* = 151), was characterized by relatively lower risk factor presence, including a greater likelihood of living in a two-parent home, lower likelihood of receiving FRL, and lower likelihood of having an incarcerated parent, and less perceived social support, including less perceived support from parents, less connectedness with peers, more social dissatisfaction with peers, and a lower likelihood of having a natural mentor in their life.

**Table 2:** Latent class analysis fit statistics

No. classes	BIC	LMR-A LRT *p*	Entropy	Smallest class %	Log-likelihood
1	12,083.103	-	-	-	− 5942.311
2	11,769.868	< 0.001	0.808	28.9	− 5683.352
3	11,785.078	0.1732	0.756	24.1	− 5588.615
4

**Table 3:** Descriptive statistics for the two-class model

	Class 1 (*n* = 351)		Class 2 (*n* = 143)	
	*M*	SD	*M*	SD
Parental support	3.77	(0.17)	3.35	(0.24)
Social connectedness	3.34	(0.31)	2.51	(0.44)
Social dissatisfaction	3.10	(0.38)	1.99	(0.20)
	**%**	***n***	**%**	***n***
Living situation				
Two parent	20.1	71	34.7	50
One parent	69.7	245	58.1	83
Other	10.2	36	7.1	102
FRL	83.8	294	75.0	107
Parent incarcerated	23.6	83	15.0	21
Special adult	80.5	283	70.4	101

Means were calculated as weighted averages. Because social dissatisfaction was reverse scored, higher mean values indicate less social dissatisfaction

### Profile Group Differences

Associations of classes with youth race/ethnicity, youth, gender, and program type are presented in Table 4. Using the *Masked Vulnerability* group as the reference class, mentees in the *Unacknowledged Resilience* group were more likely to identify as youth of color (*b* =  − 0.60, *p* = 0.010). There were no significant differences by program type, age, or gender across classes.

**Table 4:** Tests of categorical latent variable multinomial logistic regressions using the 3-step procedure

	Class 1		
	*b*	(SE)	*p*
Program type	0.21	(0.36)	0.559
Age	− 0.01	(0.04)	0.809
Gender	0.42	(0.24)	0.083
Race	− 0.60	(0.23)	0.010*

Class 2 is used as the reference class

^*^p < 0*.*05

### Adaptive Coping Outcomes Based on Profile

Profile differences in academic outcomes are presented in Table 5. There were no significant differences between the classes in adaptive coping outcomes.

**Table 5:** Differences in adaptive coping outcomes across classes

	Class 1		Class 2		*W*	df	*p*
	*b*	(SE)	*b*	(SE)			
Academic self-efficacy	1.81	(0.20)	1.72	(0.20)	2.13	1	0.1445
Academic achievement	2.65	(0.30)	2.47	(0.29)	3.77	1	0.0522
Educational expectations	1.98	(0.32)	1.86	(0.30)	3.17	1	0.0749

*p* < 0*.*05; *p* < 0*.*01; *p* < 0*.*001

## Discussion

We sought to understand differential patterns of risk factors and existing social supports among youth participating in mentoring programs across the United States and to test how these patterns, or classes, differed in their associations with youths’ adaptive coping outcomes after participating in mentoring. Additionally, we examined associations between classes and youth race/ethnicity and gender because these identities confer privilege or marginality, can lead to different experienced challenges, and thus may require different forms of support. Moreover, these identities are widely studied in the mentoring literature, with some studies suggesting male mentees benefit more than their female counterparts (DuBois et al., 2011; Raposa et al., 2019). Overall, results indicated statistically meaningful subgroups of mentees based on their reported risk factors and existing social supports upon program entry. These classes (i.e., *unacknowledged resilience*, *masked vulnerability*) mapped onto two of the four profiles included in the Dual-Axis Coping Formulation of PVEST. Neither class represented *highly vulnerable* youth nor youth with *undetermined vulnerability*, which are the two groups that are most frequently included in developmental literature comparing youth considered at-risk with those considered normative, respectively (Spencer, 2006a, b). Youth mentoring programs are considered a secondary or early intervention strategy (i.e., for those showing early signs of needing additional support); therefore, the emergence of these two classes, each representing youth with a moderate degree of vulnerability, indicates that the programs included in this study are effectively targeting youth who are expected to benefit from mentoring (Herrera et al., 2013). Although the identified classes differed in their risk factor presence and social supports, they did not differ in their associations with adaptive coping outcomes at the follow-up timepoint, when controlling for baseline coping (9–16 months after program entry). This highlights the broad applicability of mentoring as a secondary intervention for youth demonstrating a moderate degree of vulnerability. In practice, schools may refer youth to mentoring if they show early signs of needing additional support, such as attendance, academic, or behavioral concerns that do not yet warrant a more intensive or individualized intervention. Other youth serving systems, such as community-based mental health providers, Department of Family Services, or the juvenile legal system, may also refer youth to mentoring to provide additional support in coordination with other services. When used as an early intervention strategy, mentoring shows positive effects for positive developmental outcomes (Herrera et al., 2013). The only sociodemographic characteristic that was significantly associated with classes was race/ethnicity, with youth in the *Unacknowledged Resilience* group more likely to identify as Youth of Color (versus White).

Most youth fit into the *Unacknowledged Resilience* group, indicating that these were the youth most frequently targeted by or referred to mentoring programs (*n* = 351). This finding is consistent with the mentoring context because youth considered “at-risk,” or with a high-risk factor presence, are commonly targeted or referred for mentoring. However, this group of youth also had stronger social support prior to entering the program. Although we did not measure the quality of the mentee-mentor relationship, past research has shown that youth who have positive relationships prior to mentoring are better able to develop a high-quality mentoring relationship (Keller & Blakeslee, 2014; Williamson et al., 2020). The quality of this relationship is an important factor in the degree to which mentoring has a positive impact on youth (Bayer et al., 2015; DuBois et al., 2002; Rhodes, 2002; Rhodes et al., 2006). As the naming of this class indicates, and as described in the PVEST framework, the resilience of youth with this profile tends to be overlooked, and the existence of this profile is often unacknowledged (Spencer, 2006a, b). Yet, it may be that because of the supports that these youth possessed, they were able to benefit from mentoring despite their higher risk factor presence. Youth with a similar degree of risk but with less support may not benefit in the same way and require more comprehensive support or intervention. Mentoring may be erroneously considered ineffective if support is not considered alongside risk. Youth in the *Unacknowledged Resilience* group were also more likely to identify as Youth of Color. Because Youth of Color often must navigate racially discriminatory experiences and other forms of oppression, they may rely more on their supports (e.g., peers, parents, natural mentors) to cope with adversity, contributing to greater resilience (Spencer, 2006a, b). Although having a marginalized racial or ethnic identity places youth at risk for experiencing greater challenges due to systemic racism in the United States, the resilience and protective factors of these youth, in part because of the adversity that they have endured, are important to consider when designing and assigning intervention.

Fewer youth fit into the *Masked Vulnerability* group (*n* = 143). This profile of youth is also often overlooked in the developmental literature because the needs of these youth are less apparent due to a lower risk factor presence relative to the *Unacknowledged Resilience* and *Highly Vulnerable* profiles (Spencer, 2006a, b). Yet the lower social support of this group provides less of a buffer if these youth are exposed to a stressor, making them more vulnerable to adverse outcomes; thus, the addition of a mentor provides a safety net that these youth may not otherwise have and, in the current study, promoted adaptive coping. For these youth, mentoring programs may play a more preventative role. Recruitment should continue to target and expand efforts focused on youth who have a lower risk factor presence but lack social support to ensure mentoring programs reach a broad population of youth, including those who are often overlooked for other prevention and intervention. Moreover, in a research context, greater attention to youth with multiple profiles of risk and protective factor presence is needed to better understand who may benefit from mentoring and other prevention or intervention efforts. When determining which youth to target, consideration of not only risk but the presence or lack of social support is important.

PVEST is a useful framework for exploring the needs and strengths of youth but has not been used extensively when studying for whom prevention and intervention efforts are effective. Instead, youth are often categorized as highly vulnerable or normative based largely on risk factor presence, without consideration for protective factors and supports. This categorization leaves out many youths who still may need intervention, such as those with low risk factor presence and low social support. It also may paint a more negative picture of youth’s vulnerabilities without inclusion of their protective factors. Moreover, if categorization of highly vulnerable versus normative youth relies only on risk factors, findings may not be reliable as protective factor presence can impact the effectiveness of an intervention in conjunction with risk factors. The current study highlights the significance of social support for understanding the degree of vulnerability youth bring to mentoring interventions. Additionally, the person-centered approach used in this study for conceptualizing patterns of risk and support better captures the dimensional nature of such factors as well as how they may cluster together and collectively affect the effectiveness of intervention.

Additionally, our findings provide insight into the types of challenges youth are bringing to the mentoring relationship as well as the existing supports they possess. This information can be used to understand the types of services youth need, as well as the strengths that could be leveraged in their communities to promote healthy development. Most of the youth (81.2%) in this study received FRL, indicating most were experiencing some degree of socioeconomic challenge, and most did not live with both parents (75.5%), indicating a possible disruption to their primary caregiving system. That being said, we did not have enough data to understand the family dynamics within homes. Living in a single-parent or other non-traditional household structure is a risk factor in the research literature (i.e., contributes to net vulnerability) (Umberson & Thomeer, 2020), but is not necessarily an experienced challenge (i.e., does not contribute to net stress) for all youth. Nevertheless, this information can be used for tailoring services to meet the needs of young people and their families, including connecting families with resources that provide economic support and supporting youth in the distress that may result from a disrupted caregiving system. Although less frequently endorsed, 21.1% of youth reported having an incarcerated parent, with a higher likelihood among those belonging to the *Unacknowledged Resilience* group. This is significantly higher than the national average of 3.6% (Maruschak et al., 2021). Thus, increased attention and training for mentors on this need specifically is warranted. Finally, understanding the social support mentees have can allow mentors to support their mentees in building these relationships if needed given the protective effect caregivers, peers, and natural mentors can have for youth.

We did not identify youth falling within the *Highly Vulnerable* or *Undetermined Vulnerability* profiles. This finding indicates programs may not be recruiting youth with the highest degree of need or those who are not demonstrating a need for support. Although we expected to identify youth across all four profiles, it may be the case that mentoring programs do not perceive youth with a low degree of vulnerability as needing a mentor and perceive those with a very high degree of vulnerability as in need of more support than a mentor can provide. Alternatively, it is possible that mentoring programs are recruiting but not retaining these youth, or that these youth are less likely to enter programs despite recruitment efforts. However, we could not test these hypotheses with our sample. Additional research is needed to understand whether youth with a higher degree of vulnerability benefit from mentoring in the same way as those with a moderate degree of vulnerability and whether those who have a low degree of vulnerability can still reap benefits from a mentor.

## Limitations and Conclusions 

Our findings should be considered in light of some limitations. First, due to small sample sizes, we were only able to compare Youth of Color to White youth. The Youth of Color category contained youth of many different racial and ethnic identities. Although these youth may face similar challenges because being non-White in the United States increases the likelihood an individual will experience discrimination and marginalization, there is incredible diversity between racial and ethnic groups that was not captured by our Youth of Color versus White comparison. This warrants a larger follow-up study where within group differences between different racial and ethnic groups can be examined. Additionally, we did not measure what mentors did in their relationship with youth; it may be that some programs did tailor intervention to individual youth, thus attending to their needs and strengths, leading to positive outcomes across mentees from different classes. Finally, other risk and protective factors that were not included in our analyses may play a role in youth’s degree of vulnerability and the effectiveness of mentoring. Future research should explore these questions to continue unpacking for whom mentoring works based on the needs and strengths youth are bringing to the mentoring relationship and to explore which strategies may be used by mentoring programs to meet the needs of the youth they are serving.

Despite these limitations, our findings highlight the importance of attending to both risk and protective factors when considering for whom prevention and intervention efforts work. Most youth entering mentoring programs presented with a moderate degree of vulnerability; but the factors contributing to this net vulnerability varied. Mentoring programs effectively targeted youth with some degree of risk, although the presence of risk factors and social supports varied between the two classes. Despite this variability, there were no differences between groups in the degree to which they benefited from mentoring in terms of adaptive coping outcomes. Although understanding risk is important, understanding the strengths and assets that youth bring to the mentoring relationship is equally important. The protective factors youth possess provide insight into the natural supports they have that can promote their thriving and be leveraged in an intervention context to promote healthy development.

## Data Availability

The data supporting the findings of this study are not publicly available because it is the property of Big Brothers Big Sisters of America.
